# Musculoskeletal complaints among Italian X-ray technology students: a cross-sectional questionnaire survey

**DOI:** 10.1186/1756-0500-3-114

**Published:** 2010-04-24

**Authors:** Antonio Lorusso, Luigi Vimercati, Nicola L'Abbate

**Affiliations:** 1 Department of Medical and Occupational Sciences, University of Foggia, Viale Pinto, 71100 Foggia, Italy; 2 Department of Internal Medicine and Public Medicine, University of Bari, Piazza Giulio Cesare 11, 70124 Bari, Italy

## Abstract

**Background:**

There is a high prevalence of musculoskeletal disorders among healthcare professional students. Although recent studies show musculoskeletal disorders are a common problem among X-ray technologists, there are no data on these disorders among students of this healthcare profession. We have therefore estimated the prevalence of musculoskeletal complaints among a group of X-ray technology students.

**Methods:**

The students (n = 109) currently attending the 3-year X-ray technologist school at a large University in the Apulia region of Southern Italy were recruited for the study, with a 100% participation rate. A questionnaire collected data concerning personal characteristics, physical exposure during training activities, and the presence of musculoskeletal symptoms in the neck, shoulders, low back, hand/wrist and legs.

**Results:**

The prevalence of complaints in any body site over the previous 12 months was 37%. Low back pain was the most frequently reported symptom (27%), followed by neck (16%), shoulder (11%), leg (8%) and hand/wrist (5%) pain. Poor physical activity was associated with the complaints.

**Conclusions:**

Our study showed prevalence rates of musculoskeletal complaints among X-ray technology students to be somewhat high, representing about half of those found in Italian technologists. The most common musculoskeletal problem was low back pain, which had also been found in research conducted among nursing students. Our research also showed a significant association between poor physical activity and the presence of musculoskeletal disorders in young university students.

## Background

Musculoskeletal disorders (MSDs) are widespread in occurrence, with significant costs and impact on quality of life. Chronic musculoskeletal pain is reported by 1 in every 4 people in developed and underdeveloped countries [1,2]. Though the aetiology of MSDs in the general population is multifactorial, exposure to occupational risk factors make an important contribution to the occurrence of the disorders [3]. Between a quarter and a third of European workers report musculoskeletal pain, and young workers seem to be at greater risk than their older colleagues [4,5]. MSDs occur more frequently in certain occupations characterized by job tasks involving exposure to physical risk factors, as found in some healthcare professions [3].

X-ray technologists, also known as radiology technicians or radiographers, are trained health professionals responsible for performing diagnostic imaging procedures, providing an essential service in clinical healthcare. In Italy, >21,000 X-ray technologists are employed in hospitals and radiology services, whose duties are in diagnostic imaging examination, including diagnostic radiography, computed tomography, magnetic resonance imaging, and mammography. In addition to X-ray technologists, others who conduct diagnostic imaging procedures include cardiovascular technologists and nuclear medicine technologists, while sonography is performed mainly by physicians. X-ray technologist tasks include preparing the patients for the radiologic examination, positioning and immobilizing them on examination table, positioning radiographic equipment over the appropriate area of patient's body, and developing X-ray films. Thus, work tasks performed by X-ray technologists frequently involve manual handling of patients and materials. Manual handling is an important risk factor in occupational musculoskeletal disorders of healthcare workers [6-8]; for example, a high prevalence of MSDs has been reported among nurses [6-10]. Similarly, recent studies have shown occupational MSDs to be a common problem among X-ray technologists, with high prevalence rates for low back (60% to 75%), neck (20% to 39%) and shoulder (21% to 28%) problems [11-14].

Students of health professions frequently have to manual handle patients during their training activities and are at risk to MSDs just as the qualified healthcare professionals themselves. Nursing students, indeed, have high rates [15-19]. In addition, high prevalences of MSDs occur in other health professional student groups, such as medical, dental and occupational therapy students [20-22].

However, to our knowledge, no data have been reported concerning MSDs among X-ray technology students, which was therefore the object of this study.

## Methods

All the students (n = 109) currently attending the 3-year X-ray technologist school at a large University in the Apulia region of Southern Italy were recruited. The students of each year of the course were assembled by the director of the school for an appropriate lecture, set up for the survey. All accepted participation and signed the informed consent. Of these, 34 (31%), 39 (36%) and 36 (33%) were attending the first, second and third years of the course, respectively. A questionnaire, used previously in a musculoskeletal survey among X-ray technologists [14], was distributed to the participants. Two first year students were absent when the questionnaire was issued, and attended the lecture set for the third year.

The questionnaire, given in detail in ref [14], collected information on individual characteristics, physical exposure during training and the presence of musculoskeletal complaints. Individual characteristics included gender, age, height/weight, smoking and leisure time physical activity. Questions on physical workload concerned the execution of physically demanding job tasks in the course of training on the hospital wards. In particular, students were asked whether they frequently undertook tasks such as patient lifting, transferring and positioning, portable equipment handling, lead-apron wearing, and cassette or other material handling. Musculoskeletal complaints in different body regions during the past 12 months were evaluated by questions derived from the Standardised Nordic Questionnaire, translated into Italian and validated [23,24]. The body regions considered were neck, shoulders, low back, hand/wrist and legs.

Statistical associations between independent variables and symptoms in any body region were evaluated using the Student t-test for continuous variables and the chi-square test for categorical variables. Subsequently, all independent variables were included in a multivariate analysis. All analyses were conducted by means of Epi Info 3.3 software, with the level of significance being set at p < 0.05.

## Results and Discussion

All 109 students completed the questionnaire and were included in the analysis. Of these, 60 (55%) were males and 49 (45%) females. Their mean age was 22.1 years (SD 3.4), and their mean body mass index was 23 (SD 2.7). Thirty students (27%) were smokers and 55 (50%) undertook regular physical activity (>3 hours per week). As far as physical workload during training activities was concerned, all the students carried out the same tasks, rotating through the same hospital wards in each study year. This means that the physical exposure during training was similar for all the subjects. The prevalence of musculoskeletal complaints at any body site in the previous 12 months was 37%. Low back pain (LBP) was the most commonly reported symptom (27%), followed by neck (16%) and shoulder pain (11%). Table 1 shows the prevalence of the complaints with respect of the 3-year study course. Although neck, shoulder, hand/wrist and leg symptoms show a similar prevalence rate through the 3 years, low back symptoms gradually increase through the course. In Table 2 showing statistical analyses for musculoskeletal complaints at any body site, a significant association was found between poor physical activity and complaints. Multivariate analysis showed no statistical association between LBP and year of education.

**Table 1:** Prevalence of musculoskeletal complaints with respect of the three years of the study course

	Whole group		First year		Second year		Third year	
				
	n	%	n	%	n	%	n	%
Any site	40	37	9	26	18	46	13	36
Low back	29	27	6	18	10	26	13	36
Neck	18	16	5	15	6	15	7	19
Shoulder	12	11	3	9	5	13	4	11
Legs	9	8	3	9	4	10	1	6
Hand/wrist	6	5	1	3	3	8	2	6

**Table 2:** Associations between complaints at any body site and individual characteristics.

		Any MSD		No symptoms		p Value
				
		n	%	n	%	
Gender	men	21	52.5	39	56.5	0.684
	women	19	47.5	30	43.5	
Smoking		9	22.5	21	30	0.371
Regular physical activity		15	37	40	58	**0.039**
						
Age; years (mean, SD)		22.6 ± 4.0		21.9 ± 3.0		0.302
BMI; Kg/m^2 ^(mean, SD)		22.6 ± 2.6		23.2 ± 2.8		0.271

SD, Standard Deviation

To our knowledge, this is the first study to investigate musculoskeletal disorders among X-ray technology students. We estimated MSD prevalence rates among a complete group of students attending a 3-year university school. Prevalence of any MSD is lower in first-year students compared to the second- and third-year students. Furthermore, by specific body site, a difference was observed in LBP prevalence rates, with an increasing trend from the first to the last year. The difference in prevalence rates through the 3 years of the course was statistically not significant.

The results can be considered along with those of a previous investigation on Apulian X-ray technologists that used the same methodology [14], making for a direct comparison (Table 3). Musculoskeletal disorders can be seen to represent a significant concern for both X-ray technologists and students of this health profession. However, prevalence rates found among students are about half of those found in established technologists, but low back pain seems to be the most common musculoskeletal problem for both.

**Table 3:** Prevalence rates of musculoskeletal complaints found in Apulian X-ray technology students and X-ray technologists

	X-ray technology students (N = 109)		X-ray technologists (N = 203)	
		
	n	%	n	%
Any site	40	37	136	67
Low back	29	27	121	60
Neck	18	16	40	20
Shoulder	12	11	43	21
Legs	9	8	28	14
Hand/wrist	6	5	25	12

Our study shows that X-ray technology students are affected by MSDs in the same way as other health professional trainee groups, such as nursing, medical, dental and occupational therapist students [15-22]. Among them, nursing and X-ray technology students are the 2 groups that report very similar physical exposure, mainly related to manual handling of patients. For this reason, a comparison of MSD prevalence rates between these 2 groups was deemed appropriate. Prevalence of any MSD found in X-ray technology students is similar to that found in nursing students surveys carried out in Japan [15], but lower than in China [16], Australia [17] and Korea [18]. In particular, low back pain prevalence is higher than reported in Japan [15], but lower than in Australia and Korea [18,19].

Poor physical activity and complaints at any body site were statistically significantly associated, according to the findings of previous data on nursing students [18]. No other associations between MSDs and individual variables were detected in the present study. However, these results should be interpreted with caution, since the small size of the study sample was inadequate to draw statistically significant conclusions. We preferred, however, to recruit students from only one University. This ensures that all the participants performed the same kind of training activities, and consequently reported a uniform physical exposure. Indeed, each Italian University has its own program of training activities, which may be different in duration and job tasks performed by the students. The difference among Universities could have represented a confounder in our case.

## Conclusions

Overall, our study shows prevalence rates of MSDs among X-ray technology students to be somewhat high, representing about half of those found in technologists. The most common musculoskeletal problem is low back pain, in agreement with MSD research conducted among nursing students. Our research also shows a significant association between poor physical activity and the presence of musculoskeletal disorders in young university students.

## Competing interests

The authors declare that they have no competing interests.

## Authors' contributions

AL contributed to conception and design of the study, performed statistical analysis and wrote the draft of the manuscript; LV collected and interpreted the data and wrote the draft of the manuscript; NL contributed to design of the study and revised critically the manuscript. All the authors read and approved the final version of the manuscript to be published.
